# Hypercortisolism: An Under‐Recognised Pathway Linked to Multi‐System Complications

**DOI:** 10.1111/dom.71149

**Published:** 2026-08-18

**Authors:** Hernan Daniel Sacoto, Vivian A. Fonseca

**Affiliations:** ^1^ Division of Endocrinology, Diabetes and Metabolism, Department of Medicine Tulane University School of Medicine New Orleans Louisiana USA

**Keywords:** clinical physiology, endocrine therapy, glycaemic control, type 2 diabetes

## Abstract

The clinical understanding of hypercortisolism (HC) has evolved from a classic overt syndrome with florid Cushingoid features to a broader spectrum of disease that includes milder presentations, often referred to as mild autonomous cortisol secretion (MACS). This shift has lowered the threshold for screening and encouraged clinicians to consider HC even in the absence of classic physical findings, particularly in patients presenting with common features, including metabolic, cardiovascular, musculoskeletal and neuropsychiatric comorbidities. Chronic cortisol excess is associated with substantial morbidity and increased mortality through widespread effects on glucose metabolism, lipid homeostasis, cardiovascular function, blood pressure control, bone and muscle health, reproductive function, cognition and mental health. Importantly, many of these complications are frequently seen in patients with MACS, particularly in association with difficult‐to‐control diabetes and hypertension, emphasising the importance of earlier disease recognition. Recent data suggest that this abnormality may be more common than previously recognised, and its identification can have important implications for patient management. Emerging evidence may also help identify individuals at higher risk through relatively simple screening tests. Recognising this abnormality, particularly in patients with common chronic diseases that are difficult to control, represents a novel and clinically important concept that may lead to more targeted evaluation and management strategies.

## Plain Language Summary

Cortisol is a hormone secreted by the adrenal gland that works in almost every organ in the body and is essential to life. Cortisol secretion increases during illness and psychological stress and then usually goes back to normal. Its secretion is regulated by signals from the brain through other hormones. Hypercortisolism (HC) is the condition in which there is excess secretion of cortisol that is no longer adequately regulated by these signals. Severe hypercortisolism affects multiple organs and systems leading to a well‐recognized but relatively rare condition call Cushing syndrome.

Recent studies have suggested that hypercortisolism is not just the classic Cushing’s syndrome but could have milder presentations, often referred to as mild autonomous cortisol secretion (MACS). Chronic HC is associated with substantial morbidity and increased mortality through widespread effects on glucose metabolism, lipid homeostasis, cardiovascular function, blood pressure control, bone and muscle health, reproductive function, cognition, and mental health. These complications are frequently seen in patients with MACS, particularly in association with difficult‐to‐control diabetes and hypertension. In such high risk individuals simple screening tests have identified HC in over 20% of that population.

The exact cause of this abnormality is unclear, but it may have a significant impact on diabetes and hypertension that is resistant to standard therapy. Fortunately, detection is relatively easy, using well‐established screening tests that can be conducted in most clinical settings. Establishing the cause of this resistance to treatment may make it possible to consider the use of specific and precise therapy that may impact outcomes. Additional studies are needed to confirm the findings so far and help us implement recently established guidelines to recognize and treat HC early.

## 1 Introduction

Hypercortisolism (HC) is defined as a prolonged and inappropriately high increase in plasma cortisol levels, resulting from either exogenous glucocorticoid administration or abnormal endogenous cortisol overproduction.

**FIGURE 1:**
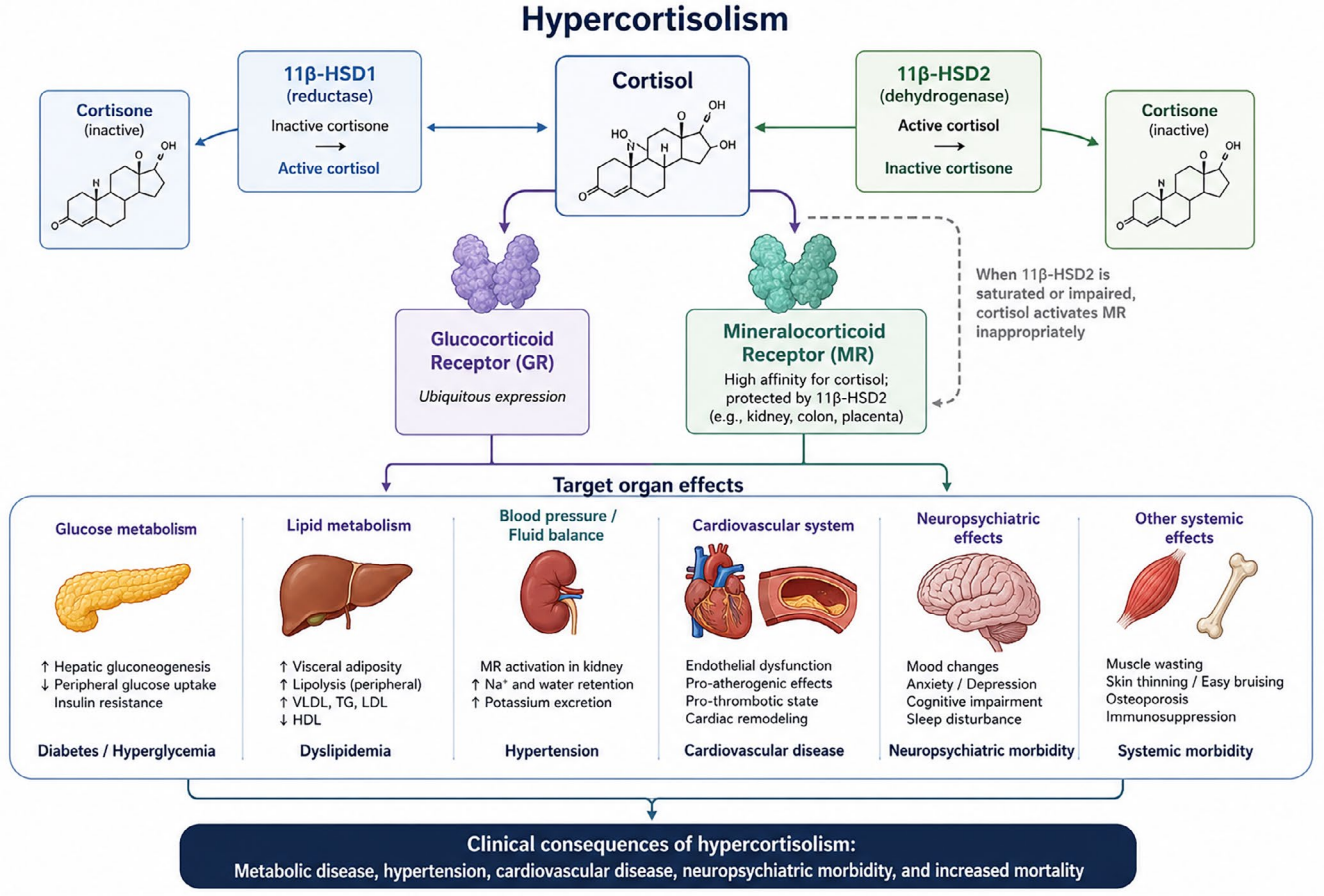
Multisystem Complications of Hypercortisolism.

HC is characterised by multisystem morbidity affecting all organ systems, resulting in metabolic derangements (hyperglycaemia, dyslipidaemia), cardiovascular complications (hypertension, hypercoagulopathy), musculoskeletal complications (osteoporosis, myopathy), immunosuppression, neurocognitive impairment and psychiatric disturbances (depression and anxiety) [1]. Figure 1 provides an overview of the multisystem complications associated with HC. The condition is associated with increased mortality if left untreated or inadequately controlled. A meta‐analysis showed that patients with endogenous HC, specifically Cushing's syndrome, had an overall standardised mortality ratio of 3.0 (95% CI, 2.3–3.9), indicating a three‐fold increased mortality risk compared to the age‐ and sex‐matched population [2]. The principal causes of death were cardiovascular disease and thromboembolism, followed by infectious complications and malignancy [2].

Our understanding of HC has evolved from viewing it as a classic syndrome characterized by severe symptoms and overt clinical features, as traditionally described in textbooks, to recognising a broader clinical spectrum that includes milder and more subtle presentations. These forms, often referred to as mild autonomous cortisol secretion (MACS), are characterised by an impaired ability to suppress cortisol production following administration of standard doses of exogenous glucocorticoids. This evolving perspective has lowered the threshold for screening and has encouraged clinicians to consider HC even in the absence of the traditional Cushingoid phenotype.

Considerable gaps in knowledge remain. Overt Cushing's syndrome does not always develop rapidly and may evolve gradually over time. As a result, clinical recognition is often delayed, particularly because the early manifestations are frequently mild and non‐specific.

Emerging data from recent clinical studies highlight that many patients with clinically significant cortisol excess lack overt physical features and instead present with common but impactful cardiometabolic conditions, such as difficult‐to‐control type 2 diabetes mellitus (T2DM) or hypertension (HTN). Recognising these subtler presentations improves detection of MACS by prompting appropriate screening. Recent evidence suggests that a more targeted screening approach may be beneficial, identifying people who would benefit from imaging to detect adenomas that may have been previously missed and potentially enabling the use of cortisol‐directed therapy in appropriately selected patients [3].

This is a narrative review. We searched databases including PubMed, Web of Science and Scopus. Our selection criteria were broad, focusing on HC, its causes, epidemiology, and its impact on various disease states and organ systems affected by the physiological effects of cortisol and its excess. Since descriptions of Cushing's syndrome date back almost a century, we did not restrict the date range, though we recognise that these databases may not have some historically important papers. In this review, we will focus on endogenous HC, its complications, and underlying pathophysiology. We will discuss the causes and epidemiology of both Cushing's disease and MACS, although the true prevalence of the latter has not been well described. Additionally, we will discuss the consequences of HC on health, with a particular focus on glucose metabolism, although there is a wide variability in clinical manifestations of the condition. Although the consequences vary considerably, and some of these, for example, mental health problems and pain, may be of importance to patients, we will focus more on the impact on glucose and BP. These conditions are highly prevalent, can be challenging to manage, and may be influenced by HC. Recognising the contribution of cortisol excess is therefore important in the evaluation and management of patients with difficult‐to‐control T2DM or HTN. Mainly because these are very common conditions that may sometimes be difficult to manage, and the impact of other disease processes may be important in the evaluation of the patient. However, it is important to recognise that not all patients with overt Cushing's syndrome have diabetes, indicating that even in this common clinical situation, expression of disease may depend on genetics and other factors that we currently do not understand.

For the purpose of this review, we define MACS as cortisol secretion that is autonomous and therefore does not respond to normal feedback suppression and is otherwise not associated with the florid features of overt Cushing's syndrome. The European Endocrine Society defined the condition based on a 1 mg dexamethasone suppression test (DST) with a plasma cortisol > 1.8 mcg/dL despite a plasma dexamethasone level > 140 ng/dL. This test is recommended in guidelines as a screening test, as it is the most sensitive. Other tests include a urine‐free cortisol and loss of diurnal variation in salivary cortisol, which may be more specific but are less sensitive. Thus, an abnormal DST may be an early abnormality present in mild cases and has been used in much of the literature on MACS. In normal individuals, following administration of 1 mg dexamethasone the previous night, the morning plasma cortisol is usually < 1.2 mcg/dL in 95% of individuals [4]. Further, a value > 1.8 mcg/dL has been associated with higher morbidity and mortality [5]. It is important to recognise that a careful history and clinical evaluation should be conducted to exclude medications or conditions that may lead to a false elevation in cortisol. Additionally, plasma dexamethasone should be measured at the time of cortisol measurement, and the test is considered invalid if the level is low. Tests are usually considered invalid if serum dexamethasone levels are below the cutoff (< 140 ng/dL) [6]. It is unclear whether these conditions were met in early studies of this condition.

## 2 Causes

The causes of endogenous HC are classified as ACTH‐dependent and ACTH‐independent. Cushing's syndrome is the general term for any condition causing high cortisol levels, while Cushing's disease is a specific type of syndrome caused by a pituitary tumour. Among ACTH‐dependent causes, Cushing's disease due to corticotropin‐secreting pituitary adenomas represents the most common form, with most cases being microadenomas (< 10 mm). Macroadenomas, 10 mm or more in diameter, account for 10% of corticotroph adenomas [7]. They are typically benign and arise from a monoclonal expansion of corticotroph cells in the anterior pituitary gland [8]. Ectopic ACTH secretion accounts for 6%–10% of overt Cushing's syndrome, arising from neuroendocrine neoplasms of pulmonary, mediastinal, pancreatic and medullary thyroid origin, some of which occur in the context of hereditary endocrine syndromes [1, 9].

Among ACTH‐independent aetiologies, unilateral adrenal adenomas account for approximately 70% of cases of overt adrenal Cushing's syndrome, while adrenocortical carcinomas constitute most of the rest. Adrenal adenomas are mostly benign and originate in the zona fasciculata of the adrenal cortex [1, 10]. In contrast to benign adrenal adenomas, adrenocortical carcinomas are characteristically larger but are far less common [11]. Other, less common aetiologies include bilateral adrenal disorders, such as primary bilateral macronodular adrenal hyperplasia, which accounts for approximately 1% of Cushing's syndrome and is sometimes associated with germline mutations. Micronodular adrenal hyperplasia represents 2% of cases and includes primary pigmented nodular adrenocortical disease, which may occur in association with Carney complex [12, 13, 14].

As discussed above, HC is not just classic Cushing's syndrome. While all patients with Cushing's syndrome have HC, not all patients with biochemical evidence of elevated cortisol meet the diagnostic criteria for Cushing's syndrome. This distinction includes other clinical entities, including MACS. While the true prevalence is not known, MACS appears to be the most prevalent form of HC and is defined by endogenous HC with post 1 mg DST cortisol greater than 1.8 mcg/dL, sometimes in the presence of an adrenal adenoma, but without overt clinical signs of Cushing's syndrome such as purple striae, easy bruising or proximal muscle weakness [15]. The clinical relevance lies in the fact that despite the absence of overt cushingoid features, it is associated with increased risk of cardiometabolic complications, vertebral fractures and increased mortality [16]. Because of the absence of overt features of Cushing's syndrome, the condition may often be missed unless screening is conducted in a targeted manner, as in recent studies [3, 17]. This form of HC was identified in several studies occurring in over 20% of people with difficult‐to‐control T2DM and even higher in those who needed three or more medications for HTN [17, 18, 19, 20]. Thus, this form of HC is far more common than the classic form of Cushing's syndrome, at least in such a population.

The term ‘non‐neoplastic HC’, sometimes referred to as ‘pseudo‐Cushing's syndrome’, refers to mild endogenous cortisol elevation caused by physiologic hypothalamic–pituitary–adrenal (HPA) axis stimulation by other disease states (e.g., severe depression or alcoholism) rather than a tumour, creating diagnostic difficulty. We prefer to use the term ‘Non‐neoplastic Hypercortisolism’ when there is a true elevation in cortisol that is not adequately suppressed with 1 mg DST and when no structural abnormalities or tumours are detectable. We use ‘pseudo‐Cushing's syndrome’ when the 1 mg DST suppression test is not conducted appropriately (plasma dexamethasone levels are low or undetectable) or the underlying conditions causing false elevation are overt. The false positive tests that lead to ‘pseudo‐Cushing's syndrome’ can be greatly minimised by careful exclusion of people with some conditions (e.g., severe psychosis or oestrogen use) and measurement of plasma dexamethasone at the time of testing, which identifies those who did not take the drug or are fast metabolizers. In contrast, non‐neoplastic HC, often associated with MACS, is now being increasingly recognised in people with chronic metabolic disease. It has been hypothesised that this clinical scenario is caused by dysregulation of the HPA axis, specifically an increased secretion of corticotropin‐releasing hormone, which stimulates ACTH secretion and subsequent adrenal cortisol production [21, 22, 23]. However, there are no prospective studies that have investigated the natural history of the development of these abnormalities.

It has increasingly been recognised that MACS is not uncommon in patients with obesity, metabolic syndrome, polyendocrine metabolic ovarian syndrome (PMOS), uncontrolled diabetes, HTN and less severe psychiatric disorders [22]. Distinguishing non‐neoplastic HC from MACS and even overt Cushing's syndrome may be difficult, as these entities share clinical and biochemical features [24]. In the CATALYST study, conditions and medications likely to lead to elevations were carefully excluded, making it unlikely that those with the abnormal 1 mg DST had ‘pseudo–Cushing's Syndrome’. However, over 70% of those with an abnormal test did not have a tumour on imaging [17].

In the setting of diabetes, MACS has been attributed to dysregulation of the HPA axis, which can be seen in chronic hyperglycaemia. It has been proposed that a vicious cycle of HC and insulin resistance triggered by metabolic stress acts through the brain to disrupt HPA feedback and ultimately chronically worsen T2DM [23, 25]. It is important to recognise that this hypothesis is difficult to prove in prospective human studies and that some of the abnormalities have been observed only in animals [26].

Nevertheless, this concept remains controversial, and further studies are needed. Another prevalence study in a similar patient population is ongoing [27]. Patients with HC will be randomised to an inhibitor of 11—beta hydroxysteroid dehydrogenase inhibitor or a placebo to determine if such an approach will improve glycaemic control. In a previous trial, an 11β‐hydroxysteroid inhibitor in uncontrolled diabetes led to a modest improvement in glycaemia, perhaps because subjects were not selected based on the presence of HC [28].

## 3 Epidemiology

The true prevalence and incidence of HC are variable due to diagnostic challenges and the under‐recognition of subclinical forms. Overt Cushing's syndrome is a rare disease, and the true population‐based prevalence has not been systematically evaluated. However, it has been estimated that the prevalence of endogenous Cushing's syndrome is estimated between 39 and 79 cases per million, with a female predominance and a female‐to‐male ratio of 4:1 [13, 29]. The incidence of new cases of endogenous Cushing's syndrome ranges from 2 to 3 cases per million people per year [13, 29, 30, 31, 32, 33, 34].

Cushing's disease (pituitary adenomas) accounts for 60%–70% of cases, adrenal causes 20%–30% and ectopic ACTH secretion accounts for 6%–10% [10, 35]. Adrenal aetiology includes unilateral adrenal tumours (adenoma or carcinoma) as well as bilateral adrenal hyperplasia, either micronodular or macronodular [14, 24, 32, 35, 36, 37].

Among patients with adrenal incidentalomas, which are present in 1%–7% of the adult population, MACS can be identified in around 20% of cases, yielding an estimated population prevalence of 0.2%–3.5%, substantially higher than that of overt Cushing's syndrome [15, 16].

However, these figures do not capture MACS without a presentation of adrenal incidentaloma, which may be more prevalent, as discussed above. Most studies evaluating MACS are small and retrospective in nature, limiting the accuracy of prevalence estimates. Nevertheless, a high prevalence of MACS has been reported in populations with difficult‐to‐manage conditions that may be exacerbated by cortisol excess, particularly diabetes and HTN [18, 19, 20]. About 20%–30% of these patients have an abnormality on adrenal imaging.

In the prospective CATALYST study of 1000 patients with difficult‐to‐treat T2DM, HC was identified in 23% of participants. The prevalence increased to over 30% among individuals requiring more than three antihypertensive agents, highlighting the potential contribution of cortisol excess to treatment‐resistant cardiometabolic disease [3, 17]. Consistent with these findings, the ongoing CAPTAIN‐T2D trial is evaluating the prevalence of HC in a similar population of patients with difficult‐to‐control T2DM [27]. Further supporting these observations, the ongoing multicentre MOMENTUM study is assessing the prevalence of endogenous HC in adults with resistant HTN, some of whom also have diabetes. Preliminary results presented at the recent American College of Cardiology Scientific Sessions 2026 revealed a similarly high prevalence of HC, suggesting that cortisol excess may be substantially underrecognised in this high‐risk population, and found a similar prevalence [38, 39, 40].

## 4 Consequences and Clinical Significance

Hypercortisolaemia is a multisystem clinical syndrome that affects virtually every organ system either directly or indirectly, resulting in substantial morbidity and increased mortality [36, 37] The more common manifestations of HC are summarised in Figure 1. It is important to recognise that the complications are closely linked with HC and may coexist at a rate that is higher than expected. For example, cardiovascular events (myocardial infarction, stroke) and skeletal fragility (osteoporosis, fractures) are both significantly elevated even before diagnosis [1, 37, 41].

### 4.1 Metabolic Complications

The metabolic complications associated with HC are summarised in Table 1.

**TABLE 1:** Summary of the metabolic complications of HC.

Conditions	Prevalence/characteristics	Key mechanisms	Clinical implications
Glucose metabolism [37]	– Impaired fasting glucose (7%–64%) – Impaired glucose tolerance (6%–14%) – Type 2 diabetes mellitus (11%–47%)	– Insulin resistance and β‐cell dysfunction [42] – ↑ Gluconeogenesis [43] – ↓ Glucose Transporter Type 4 and ↑ lipolysis [44, 45]	– Poor glycaemic control
Dyslipidaemia	– ↑ total cholesterol, LDL, triglycerides; variable HDL [46]	– ↓ Lipoprotein lipase, impaired VLDL clearance [44, 47] – ↑ ApoB and LDL particles [48]	– Atherogenic profile → ↑ cardiovascular risk
Visceral adiposity	– Common phenotype in HC	– Central fat redistribution – ↑ Lipolysis and free fatty acids – ↑ Local 11β‐hydroxysteroid dehydrogenase type 1 (11β‐HSD1) activity [49, 50]	– Insulin resistance and cardiometabolic risk
Hepatic steatosis (MASLD)	Up to 66% in cushing's syndrome and 57% in MACS [51, 52, 53]	– ↑ Free fatty acids flux, hepatic lipogenesis, and insulin resistance – ↑11β‐HSD1	– ↑ Fat deposition and fibrosis risk

#### 4.1.1 Impaired Glucose Metabolism

Chronic glucocorticoid excess has widespread effects across different tissues involved in glucose homeostasis, including the liver, skeletal muscle, adipose tissue and pancreatic islets. The aggregated prevalence data reported by Pivonello et al. showed that abnormalities in glucose metabolism occur in 27%–87% of patients with Cushing's syndrome, including impaired fasting glucose (7%–64%), impaired glucose tolerance (6%–14%) and T2DM (11%–47%) [37]. Diabetes prevalence is higher in ectopic ACTH secretion (74%) than in pituitary (33%) or adrenal causes of HC (34%) [35, 37]. Recent studies also demonstrate a high prevalence of MACS in patients with difficult‐to‐control T2DM despite complex treatment regimens [3, 17, 54, 55].

Impaired glucose metabolism in HC results from various disruptions in glucose homeostasis involving both peripheral insulin resistance and impaired pancreatic β‐cell function. This is thought to be the result of a mechanism involving genomic and non‐genomic glucocorticoid receptor (GR) pathways [37, 42]. In the liver, glucocorticoids upregulate gluconeogenic enzymes, leading to increased hepatic glucose production [43, 56]. In skeletal muscle and adipose tissue, glucocorticoids impair glucose utilisation by promoting lipolysis and therefore increasing circulating free fatty acids, which contribute to peripheral insulin resistance, while also inhibiting insulin‐stimulated translocation of glucose transporter type 4 to the plasma membrane [43, 44, 45]. Additionally, glucocorticoids exert direct toxic effects on pancreatic β‐cell mass and function [45, 57, 58]. While some of these studies have been done in animals and may therefore have only theoretical implications, Rizza et al. conducted an interesting human experiment. Healthy subjects were given a low dose of dexamethasone for a short duration and rapidly developed impaired insulin action as well as a possible reduction in insulin secretion [57].

#### 4.1.2 Dyslipidaemia

HC is characterised by increased total cholesterol, LDL cholesterol and triglyceride levels, with variable alterations in HDL cholesterol [37, 46]. Hypertriglyceridemia in chronic glucocorticoid exposure results from reduced adipose tissue lipoprotein lipase activity and impaired VLDL removal [47]. Nuclear magnetic resonance spectroscopy demonstrated that HC is associated with elevated LDL particle concentrations and apolipoprotein B levels; clinically, this may translate into increased cardiovascular morbidity and mortality [48]. However, prospective studies of the consequences of dyslipidaemia in this clinical situation are not available. Unlike in diabetes and HTN, there are no studies suggesting that HC has difficulty in the clinical management of this condition.

#### 4.1.3 Central Obesity and Liver Steatosis

Central adiposity is a key feature of both Cushing's syndrome and MACS. However, obesity itself assessed with a high body mass index alone does not predict HC [17]. It is well established that even a moderate degree of central obesity is associated with fat deposition in abdominal organs [37, 51]. Metabolic dysfunction associated steatotic liver disease (MASLD) can be seen in up to 66% of patients with Cushing's syndrome and in up to 57% MACS cases [51, 52, 53].

The visceral adiposity characteristic of HC may lead to increased activity of 11β‐hydroxysteroid dehydrogenase type 1 (11β‐HSD1) in adipose tissue, which converts inactive cortisone into active cortisol. This creates a local amplification of glucocorticoid exposure within adipose deposits, perpetuating fat accumulation and hepatic steatosis [49, 50].

The pathophysiology of liver steatosis involves molecular mechanisms, insulin resistance, visceral adiposity and altered adipokine secretion [37, 44, 51, 59]. Glucocorticoids induce lipolysis in adipose tissue, especially visceral fat, resulting in elevated circulating free fatty acids that are taken up by the liver and serve as substrates for triglyceride and VLDL, contributing to hepatic steatosis [37, 44, 59]. Successful treatment of HC is associated with remission of hepatic steatosis, with up to 75% of patients with MASLD at diagnosis demonstrating resolution within 1–5 years following remission of Cushing's syndrome [52]. In the CATALYST study, treatment decreased waist circumference significantly, though liver fat changes were not quantified [55].

### 4.2 Cardiovascular Complications

Cardiovascular complications are among the leading causes of morbidity and mortality in patients with HC. Cardiovascular complications associated with HC are summarised in Table 2. The high rates of cardiovascular events in these patients are probably related to abnormal lipid metabolism, HTN, cardiac remodelling, dysfunction and vascular atherosclerosis [68].

**TABLE 2:** Summary of cardiovascular complications of HC.

Complications	Key mechanisms	Clinical implications
Hypertension (HTN)	– Saturation of 11β‐hydroxysteroid dehydrogenase type 2 → inappropriate mineralocorticoid receptor (MR) activation [49, 60, 61, 62, 63] – ↓ Nitric oxide [64, 65].	– Resistant HTN [66, 67].
Atherosclerosis/coronary artery disease	– Dyslipidaemia and HTN. Endothelial dysfunction: ↓ NO [63, 64, 65]. – Insulin resistance and dyslipidaemia lead to ↑ arterial stiffness [59]	– ↑ Cardiovascular events [41]
Myocardial infarction (MI)	Linked to cardiometabolic risk factors and direct myocardial cortisol effects via MR/glucocorticoid receptor (GR) [49, 68]	– High prevalence MI [2, 37, 41]
Heart failure	– Left ventricle hypertrophy and concentric remodelling [49, 68].	– Systolic/diastolic dysfunction [68, 69, 70]
Stroke	– Accelerated atherosclerosis, endothelial dysfunction, prothrombotic state.	– ↑ Stroke risk [24, 37, 71].
Venous thromboembolism (VTE)	– Hypercoagulable state [71, 72]. – Impaired fibrinolysis [73].	– ↑ VTE risk [24, 37, 71]

#### 4.2.1 Hypertension

Hypertension (HTN) is present in up to 82% of patients with Cushing's syndrome and 64% of patients with MACS [74]. It can be the result of direct and indirect mechanisms acting on the kidney, vasculature and central nervous system. A central mechanism involves the saturation of 11β‐hydroxysteroid dehydrogenase type 2 (11β‐HSD2), the enzyme that normally inactivates cortisol by converting it to cortisone before it can bind to the mineralocorticoid receptor (MR) in the kidney. When 11β‐HSD2 is oversaturated by excess cortisol, the MR is inappropriately activated, leading to increased sodium and water retention [49, 60, 61, 62, 63]. Glucocorticoids can also impair endothelium‐dependent vasodilation and induce excess superoxide production in vascular endothelial cells, leading to a reduction in nitric oxide bioavailability [64, 65]. These alterations may result in persistent HTN despite remission of HC [66, 67].

The prevalence of HC in patients with resistant HTN was evaluated in the MOMENTUM trial discussed above, which was also discussed in a recent cardiology review paper by Einhorn et al. [39, 40]. In a small open‐label trial of a selective GR modulator relacorilant, improvements in BP and hyperglycaemia were observed. Systolic and diastolic BP (SBP, DBP; assessed by ambulatory blood pressure monitoring) were significantly reduced over 22 weeks (mean change ± standard deviation SBP: −7.9 ± 9.8 mmHg, DBP: −5.4 ± 7.0 mmHg; *p* < 0.0001) [75]. This approach needs to be evaluated in patients with MACS.

#### 4.2.2 Myocardial Infarction and Heart Failure

Myocardial infarctions are known to occur more frequently in patients with Cushing's syndrome [2, 37, 41]. This increased cardiovascular risk is thought to be mediated, in part, by aberrant cardiac remodelling and dysfunction, as myocytes express both MR and GR but lack 11β‐HSD2; consequently, supraphysiological cortisol levels can activate both receptors [49, 68].

Clinically, HC can present with systolic and diastolic dysfunction, left ventricular concentric remodelling and hypertrophy, and less commonly, dilated cardiomyopathy. Importantly, HC‐associated cardiomyopathy has been described even in patients without HTN, suggesting that cardiac remodelling is not solely driven by elevated blood pressure but also by direct effects of cortisol on the myocardium [68, 69, 70].

#### 4.2.3 Stroke and Venous Thromboembolism (VTE)

These conditions represent major contributors to morbidity and mortality in patients with HC, with stroke risk increased by a hazard ratio of 4.5 (95% CI 1.8–11.1) and VTE risk elevated 10‐ to 18‐fold compared with the general population, with an incidence rate of 14.6 per 1000 person‐years (95% CI 10.3–20.1) [24, 37, 71]. Hypercoagulability in HC is largely driven by changes in the intrinsic coagulation pathway, a rise in the number of platelets, reflecting an overall prothrombotic state [72, 73]. Impaired fibrinolysis is another key contributor to the prothrombotic state in HC [76].

### 4.3 Musculoskeletal Complications

The musculoskeletal complications associated with HC are summarised in Table 3.

**TABLE 3:** Summary of musculoskeletal complications of HC.

Complications	Key mechanisms	Clinical implications
Bone disease	– ↑ Osteoclast activity, ↓ Osteoblast differentiation [77, 78, 79, 80, 81]. – Impaired bone quality [77, 78, 79, 80]	– Fragility fractures (vertebral) [16, 81]
Myopathy	– ↓ Protein synthesis: myogenesis, ↑ Proteolysis, ↑ Myostatin [82, 83, 84]	– Proximal muscle weakness [82, 83]

#### 4.3.1 Osteoporosis

Bone disease in HC is a well‐recognised cause of secondary osteoporosis. Among patients with chronic glucocorticoid exposure, the prevalence of osteoporosis is estimated to be as high as 50% [81]. Patients with MACS also have an increased prevalence of osteoporosis and its complications, including fragility fractures, reduced quality of life, and increased mortality [16, 77]. Vertebral fracture risk is not directly correlated with the degree of bone mineral density loss but rather with impaired bone quality [77, 78, 79].

The pathogenesis of osteoporosis involves increased bone resorption through enhanced osteoclast activity. Glucocorticoids promote osteoclast differentiation and survival by increasing the availability of receptor activator of nuclear factor kappa‐B ligand from osteoblasts and osteocytes and by reducing osteoclast apoptosis. In addition, glucocorticoids impair bone formation by inhibiting differentiation of stromal cells into osteoblasts [80, 81].

#### 4.3.2 Myopathy

Myopathy is a common manifestation of HC [82, 83]. Steroid myopathy involves anti‐anabolic and catabolic mechanisms and is characterised by muscle weakness predominantly affecting the proximal part of the lower limbs [84]. Steroid myopathy significantly affects quality of life and functional capacity. Older age, higher waist‐to‐hip ratio and baseline HbA1c were associated with worse outcomes, and persistent muscle weakness was strongly linked to reduced quality of life [85].

### 4.4 Reproductive and Sexual Dysfunction

The reproductive and sexual dysfunction associated with HC is summarised in Table 4.

**TABLE 4:** Summary of reproductive and sexual dysfunction complications of HC.

Complications	Key mechanisms	Clinical implications
Hypogonadism	– Suppression of gonadotropin‐releasing hormone (GnRH) and luteinising hormone (LH) secretion [86, 87, 88] – ↓ LH receptor expression, ↓ testosterone levels [89]	– Decreased libido, erectile dysfunction [29, 85, 86, 90]
Menstrual dysfunction/gonadal changes	– Suppression of hypothalamic–pituitary–gonadal axis [87, 88, 91] – Effects via GR on gonadal cells	– Amenorrhea, oligomenorrhea, impaired fertility [87, 90]
Central (HPG axis) suppression	↓ GnRH gene expression [87, 88, 91].	Hypogonadotropic hypogonadism

Endogenous HC, particularly Cushing's syndrome, is associated with reproductive dysfunction, including decreased libido and hypogonadism in men and menstrual irregularities in women, the latter being more common in Cushing's disease [35, 86, 87, 90].

The pathogenesis is characterised by multilevel inhibition of the hypothalamic–pituitary–gonadal axis. While local dysregulation may occur in pituitary disease, glucocorticoids directly suppress gonadotropin‐releasing hormone through downregulation of its gene expression [88, 91, 92].

At the gonadal level, glucocorticoids directly impair function through GR‐mediated suppression of sex steroid hormone synthesis and promotion of cellular apoptosis.

In men, glucocorticoids disrupt testicular steroidogenesis by suppressing luteinising hormone (LH) secretion, downregulating LH receptor expression on Leydig cells, and promoting Leydig cell apoptosis [89].

The metabolic consequences of HC further contribute to reproductive dysfunction. Increased visceral adiposity and hepatic steatosis are associated with dysregulated sex steroid metabolism, reduced sex hormone binding globulin concentrations and androgen excess [37, 86].

### 4.5 Neuropsychiatric Complications, Cognitive Dysfunction

Neuropsychiatric complications and the cognitive dysfunction associated with HC are summarised in Table 5.

**TABLE 5:** Summary of neuropsychiatric complications and cognitive dysfunction in HC.

Complications	Key mechanisms	Clinical implications
Neuropsychiatric disorders (depression, anxiety, bipolar)	– Structural and functional changes in GR‐rich brain regions: hippocampus, amygdala, prefrontal cortex [37, 93]	– Major depression most common (up to 81%), anxiety (66%) and bipolar disorders (30%) [94] – Also observed in MACS in 25% vs. 11% in nonfunctioning incidentalomas [95]
Cognitive impairment	– Reduced grey matter volume, cortical thickness, ventricular enlargement, white matter disruption [96].	– Impairment in working memory, executive function, attention, short‐term memory [97, 98] – Mild cognitive impairment in MACS [99]

Neuropsychiatric manifestations represent some of the most common and debilitating complications of Cushing's syndrome, which may improve but not completely remit on treatment. Studies in patients with Cushing's syndrome and Cushing's disease have identified major depression as the most prevalent psychiatric disorder (up to 81%), followed by anxiety disorders (66%) and bipolar disorders (30%) [94]. Psychiatric manifestations are also seen in MACS, with anxiety and depression reported in up to 25% of patients compared with 11% in nonfunctioning adrenal incidentalomas [95]. It is clinically important to highlight this complication since major depression may be the initial manifestation of endogenous HC in some patients [37, 93]. In the CATALYST study, approximately 28% of participants were taking analgesics, and 27% were taking psychiatric medications (usually antidepressants), although patients with severe psychiatric disease were excluded from the study [3].

Chronic cortisol excess leads to structural and functional changes in key brain regions rich in GR, particularly the hippocampus, amygdala and prefrontal cortex, therefore affecting cognition and emotional regulation [37, 97]. Several abnormalities on neuroimaging studies have been demonstrated in patients with Cushing's disease, including reduced grey matter volume and cortical thickness, along with ventricular enlargement, cerebral atrophy and widespread white matter disruption [96].

Cushing's disease is associated with cognitive dysfunction, involving impairments in working memory, executive function, attention and short‐term memory [98]. Patients with MACS have a higher prevalence of subjective memory complaints and mild cognitive impairment compared with individuals with nonfunctioning adrenal adenomas [99].

### 4.6 Dermatological Complications

Cutaneous manifestations of HC include wide purple striae (> 1 cm), facial plethora, easy bruising, skin atrophy, impaired wound healing, hyperhidrosis, hyperpigmentation, acanthosis nigricans, acne, hirsutism and alopecia [37, 83].

When distinguishing HC from other metabolic conditions, such as metabolic syndrome or PMOS, certain skin findings are more specific. Petechiae and ecchymoses arise from increased vascular fragility and thinning of the dermis and epidermis secondary to collagen loss. Facial plethora is attributed to polycythaemia and is frequently associated with facial telangiectasias [100].

Hair changes, including hirsutism, acne and alopecia, are driven by hyperandrogenism, present in up to 50% of cases, especially in ACTH‐dependent HC [101]. Predominance of hirsutism may lead to a diagnosis of PMOS being made without considering HC.

## 5 Conclusion

HC is associated with a wide range of common clinical disorders, such as diabetes and HTN. The exact cause of this abnormality is unclear, particularly in individuals, but its impact on glucose and blood pressure makes these conditions difficult to treat and resistant to standard therapy. Fortunately, detection is relatively easy, using well‐established screening tests that can be conducted in most clinical settings. Establishing the cause of this resistance to treatment may make it possible to consider the use of specific and precise therapy that may impact outcomes. Additional studies are needed to confirm the findings so far and help us develop guidelines to recognise and treat HC early. In this context, recently, an Italian Position Statement has been published, and an Endocrinology guideline for diabetes recommends screening for HC and its management in patients who are difficult to control [102, 103]. Ongoing clinical trials may help guide us further on the appropriate screening and management of this condition.

## Funding

The authors have nothing to report.

## Disclosure

Dr. Fonseca has declared: Research Support (to Tulane): Grants from Novo Nordisk, Sparrow, Alnylam, Immunovant. Honoraria for Consulting and Lectures: Regeneron, Abbott, Corcept, Eli Lilly, Boehringer Ingelheim. Stock Options: Mellitus Health, BRAVO4Health. ‐Stock Taysha Gene Therapy. Potential conflicts of interest did not influence the development of this review.

## Conflicts of Interest

The authors declare no conflicts of interest.

## Data Availability

Data sharing not applicable to this article as no datasets were generated or analysed during the current study.
